# Integrale domeinoverstijgende samenwerking voor zwangere vrouwen en (aanstaande) ouders in een kwetsbare situatie: Basisstructuur Kansrijke Start Zuid-Limburg

**DOI:** 10.1007/s12508-025-00458-0

**Published:** 2025-03-25

**Authors:** Nicole Smeets-Curvers, Marijke Hendrix, Darie Daemers, Rowan Drittij, Birgit Levelink, Mandy Stijnen

**Affiliations:** 1 Afdeling Kennis & Innovatie, GGD Zuid-Limburg, Academische Werkplaats Publieke Gezondheid Mosa (AWPG Mosa), Heerlen, Nederland; 2 Vakgroep Sociale Geneeskunde, Care And Public Health Research Institute (CAPHRI), Faculty of Health Medicine and Life Sciences (FHML), Universiteit Maastricht, Maastricht, Nederland; 3 Lectoraat Midwifery Science, Academie Verloskunde Maastricht (AVM), Zuyd Hogeschool, Maastricht, Nederland; 4 Kansrijke Start Parkstad, Gemeente Heerlen, Heerlen, Nederland; 5 Afdeling Kindergeneeskunde – sociale pediatrie, Mosakids Kinderziekenhuis, Maastricht UMC+, Maastricht, Nederland

**Keywords:** samenwerking, Kansrijke Start, zwangeren, (aanstaande) ouders, kwetsbaarheid, geboortezorg, jeugdgezondheidszorg, gemeenten, Collaboration, Solid Start, Pregnant women, Parents, Expectant parents, Vulnerability, Maternity care, Youth health care, Municipalities

## Abstract

Integrale domeinoverstijgende samenwerking wordt gezien als de sleutel tot succes om complexe uitdagingen in de gezondheid en zorg voor zwangere vrouwen en (aanstaande) ouders het hoofd te bieden. Zuid-Limburg werkt aan het inrichten en optimaliseren van de samenwerking tussen het medisch, sociaal en publiek gezondheidsdomein. Het resultaat is een regionale Basisstructuur Kansrijke Start voor het bieden van passende steun aan gezinnen in een kwetsbare situatie. Dit artikel schetst de ontwikkeling, opzet en geleerde lessen van deze Basisstructuur.

**Digitaal aanvullende content:**

De online versie van dit artikel (10.1007/s12508-025-00458-0) bevat aanvullend materiaal, toegankelijk voor daartoe geautoriseerde gebruikers.

## Abstract (en)

Integrated cross-sectoral collaboration is seen as the key to success in meeting complex challenges in the health of and care for pregnant women, parents, and expectant parents. South Limburg focuses on establishing and optimizing the collaboration between the medical, social, and public health sectors. The result is a regional Solid Start Integrated Approach for providing appropriate support to families in a vulnerable situation. This article describes the development, design, and lessons learned of this integrated approach.

## Aanleiding

Zuid-Limburg is een van de regio’s in Nederland die al jaren achterstanden kent in gezondheid en participatie. Inwoners van Zuid-Limburg leven korter en minder jaren in goede gezondheid [1]. Een belangrijke verklarende factor is het lage opleidingsniveau en een lagere sociaaleconomische positie van de inwoners [1, 2]. Vaak ontstaat de achterstand al aan het begin van het leven en gaat deze gepaard met een groter risico op vroeggeboorte, een laag geboortegewicht en gezondheids- en ontwikkelingsproblemen bij kinderen [3, 4].

Ondanks een gestage vooruitgang over de jaren heen worden in Zuid-Limburg nog steeds meer kinderen te vroeg en/of met een laag geboortegewicht geboren dan in de rest van Nederland. Dit probleem werd niet alleen gezien door geboortezorgprofessionals (zoals verloskundigen, gynaecologen, kinderartsen), maar kwam in 2018 ook bij gemeenten in beeld via regionale en gemeentelijke cijfers over vroeggeboorte en/of een laag geboortegewicht. Als eerste regio in Nederland vormde Zuid-Limburg daarom eind 2018 een Regionale Coalitie Kansrijke Start (hierna ‘Regionale Coalitie’) tussen samenwerkingspartners uit het medisch, sociaal en publiek gezondheidsdomein (zie bijlage 1 in de digitaal aanvullende content), met als gezamenlijke ambitie om meer kinderen een kansrijke start te geven. De ambitie landde ook in het regionaal gezondheidsbeleid 2020–2023 van de zestien Zuid-Limburgse gemeenten. In deze nota is Kansrijke Start een van de levensfasen van het meerjarige, regionale programma Trendbreuk, dat bedoeld is om de gezondheidsachterstand van Zuid-Limburg ten opzichte van Nederland in 2030 met 25% in te lopen [5]. In lijn met het landelijke actieprogramma Kansrijke Start is in de daaropvolgende jaren de regionale samenwerking verder uitgebouwd. Dit is mogelijk gemaakt vanuit een eigen bijdrage van partners in de vorm van personele inzet en met externe financieringsbronnen. Het Gezond en Actief Leven Akkoord (GALA) en de Brede SPUK-regeling (een specifieke uitkering) hebben vanaf 2023 gezorgd voor verdere beleidsmatige en financiële verankering van de ketenaanpak Kansrijke Start.

## Doel en doelgroep

De focus ligt op Kansrijke Start: de periode vanaf de (pre)conceptie tot aan het tweede levensjaar van het kind. Een belangrijk doel van Kansrijke Start Zuid-Limburg is het versterken van de regionale samenwerking tussen partners uit het medisch, sociaal en publiek gezondheidsdomein rond vroegsignalering van psychosociale risico’s en het leveren van steun op maat aan zwangere vrouwen en (aanstaande) ouders in een kwetsbare situatie.

In Zuid-Limburg is het risico op kwetsbaarheid onder (potentiële) ouders verhoogd [6]. Kwetsbaarheid wordt in Zuid-Limburg, conform de definitie van het Erasmus MC/de gemeente Rotterdam [7], gezien als een disbalans tussen risicofactoren en beschermende factoren (zie fig. 1). Tabel 1 geeft een overzicht van mogelijke kwetsbaarheden en beschermende factoren, gebaseerd op eerder onderzoek [7, 8]. De definitie gaat uit van vier categorieën van kwetsbaarheid: zelfredzaam, potentieel kwetsbaar, kwetsbaar en zeer kwetsbaar [7]. Hoewel de focus in de Rotterdamse definitie ligt op de zwangere, interpreteren we dit binnen de Basisstructuur Kansrijke Start breder en nemen we ook de partner en de fase na de zwangerschap mee.

**Figuur 1:**
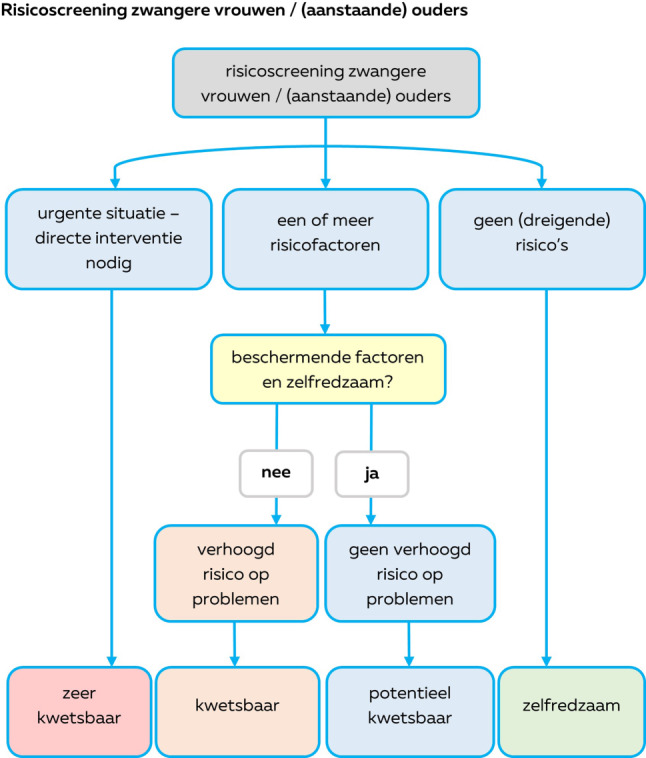
Toelichting op de definitie kwetsbaarheid gehanteerd binnen de Basisstructuur Kansrijke Start Zuid-Limburg [7]

**Tabel 1:** Overzicht van mogelijke (niet-urgente) kwetsbaarheden en beschermende factoren op basis van de definitie van kwetsbaarheid die gehanteerd wordt binnen de Basisstructuur Kansrijke Start Zuid-Limburg

kwetsbaarheden		beschermende factoren
urgente kwetsbaarheden	kwetsbaarheden	
huiselijk geweld	financiële problematiek	stabiel gezinsklimaat
betrokkenheid Veilig Thuis	suboptimaal zorggebruik	stabiele woonsituatie
betrokkenheid Raad van de Kinderbescherming	voorbereiding op zwangerschap, ouderschap/komst kind	ontvangen warmte en affectie van de eigen ouder
middelenmisbruik/verslaving (alcohol, drugs)	ongezonde leefstijl: voeding, bewegen, middelengebruik	praktische en emotionele steun vanuit het sociale netwerk
acute psychiatrische/psychische problematiek	medische factoren: medicatie, behandeling, doorverwijzing	benodigde professionele ondersteuning is aanwezig
dak- of thuisloos	laag opleidingsniveau	gezondheidsvaardigheden
	alleenstaande moeder	bereid hulp te aanvaarden
	problemen naar aanleiding van samengesteld gezin	probleemoplossend vermogen
	relatieproblemen	voldoende opvoedvaardigheden
	niet-acute psychiatrische/psychische problematiek	sociaal-emotionele vaardigheden
	tienerzwangerschap	motivatie
	ongepland en ongewenst zwanger	veerkracht ouder(s)
	woonomgeving (onveilig/ontoereikend)	welzijn/welbevinden ouder(s)
	problematiek partner (psychosociaal, verslaving of chronische aandoening)	
	taalbarrière	
	laaggeletterd en/of onvoldoende gezondheidsvaardigheden	

Om de regionale samenwerking te versterken zijn initiatieven gestart om met professionals uit het medisch, sociaal en publiek gezondheidsdomein te werken aan een betere signalering, doorgeleiding en ondersteuning voor de doelgroep. Met impulsgelden (ZonMw) en provinciale gelden is vanaf 2019 geïnvesteerd in netwerkbijeenkomsten, kennisdeling en ontmoetingen tussen professionals, managers en bestuurders uit de drie domeinen om elkaar (beter) te leren kennen, ambities en belangen te verkennen, samenwerkingsafspraken te maken en kansrijke ideeën te identificeren. Vanuit een tweetal onderzoeksprojecten zijn nieuwe werkwijzen ontwikkeld en geïmplementeerd (zie kader 1). Daarnaast is geïnvesteerd in de uitrol en borging van de interventies VoorZorg, Stevig Ouderschap en Nu Niet Zwanger. Geïnspireerd door de werkwijze van Goede Start in de Veenkoloniën kwam de Regionale Coalitie in 2022 tot het voorstel om de ontwikkelde tools, initiatieven en samenwerkingsafspraken meer in samenhang te integreren tot een regionale Basisstructuur Kansrijke Start Zuid-Limburg (hierna: Basisstructuur) (zie fig. 2; [9]).

**Figuur 2:**
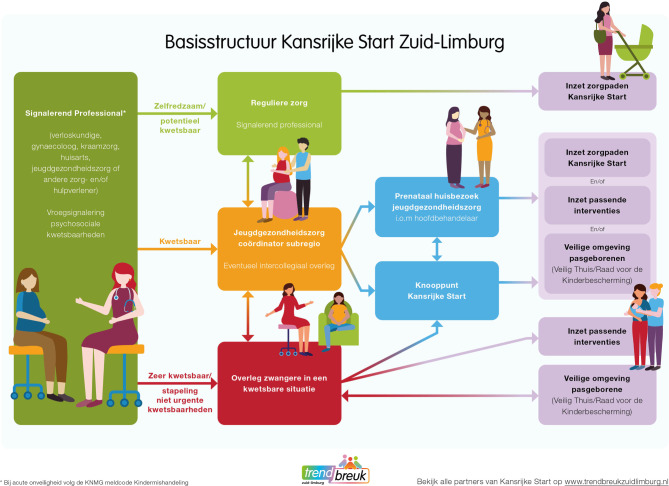
Schematische weergave Basisstructuur Kansrijke Start Zuid-Limburg

### Kader 1 Toelichting onderzoeksprojecten gekoppeld aan de regionale Basisstructuur Kansrijke Start Zuid-Limburg

*Project*: Samen voor Gezondheid (SvG I, 2020–2023; SvG II, 2022–2026)

*Initiatiefnemer*: Academie Verloskunde Maastricht (AVM)

*Financiering*: Regieorgaan SIA (RAAK.PUB06.007) en ZonMw (05430052110001)

*Doel*: samen met professionals uit de geboortezorg en het sociaal domein kennis ontwikkelen, delen en duurzaam implementeren van twee verschillende aanpakken gericht op de zorg voor kwetsbare zwangeren: 1) het identificeren van kwetsbare zwangere vrouwen met een digitale psychosociale vragenlijst en 2) het organiseren van een goede doorgeleiding naar en samenwerking met het sociaal domein via één persoonlijk aanspreekpunt bij de jeugdgezondheidszorg [8, 10].

*Project*: Samenhangende zorg voor kwetsbare zwangeren en kwetsbare jonge ouders: Knooppunten Kansrijke Start in Zuid-Limburg (looptijd 2021–2025)

*Initiatiefnemer*: GGD Zuid-Limburg/Academische Werkplaats Publieke Gezondheid Mosa (AWPG Mosa)

*Financiering*: ZonMw (554001011)

*Doel*: het opzetten en uitvoeren van een Knooppunt Kansrijke Start: multidisciplinair overleg op gemeenteniveau tussen partijen uit het medisch domein en sociaal domein die zorg en hulp bieden aan zwangere vrouwen, jonge gezinnen en het jonge kind. Partijen zijn onder andere de verloskundige, kraamzorg, jeugdverpleegkundige, huisarts, gemeente en maatschappelijk werk. Zij gaan met de (aanstaande) ouder(s) om tafel zitten en maken afspraken over de steun die het beste aansluit bij de hulpvraag.

## Basisstructuur Kansrijke Start Zuid-Limburg

De doelen van de Basisstructuur zijn:

1. zwangere vrouwen en (aanstaande) ouders in een kwetsbare situatie beter in beeld krijgen en zo snel mogelijk doorgeleiden naar de best passende ondersteuning;
2. domeinoverstijgende samenwerking tussen professionals in de fase Kansrijke Start verbeteren door heldere en uniforme regiobrede afspraken;
3. waarborgen van gelijke toegang tot passende ondersteuning voor alle ouders.

In de Basisstructuur staat de eerder genoemde definitie van kwetsbaarheid centraal (zie tab. 1 en fig. 1; [7, 8]). Afhankelijk van de mate van kwetsbaarheid worden verschillende ondersteuningsopties aangeboden. De Basisstructuur toont de route van signaleren tot steun op maat. Concrete werkafspraken en bijbehorende formulieren zijn uitgewerkt in een onderliggende *Handleiding Kansrijke Start Zuid-Limburg*. Hieronder volgt een toelichting op de onderdelen van de Basisstructuur.

### Signalerend professional

Verloskundig zorgverleners, zoals verloskundigen en gynaecologen, hebben een belangrijke rol bij de vroegsignalering van kwetsbaarheden tijdens de zwangerschap. Eerstelijnsverloskundigen in Zuid-Limburg werken niet met een bestaand, gevalideerd signaleringsinstrument omdat ze dit onvoldoende vinden aansluiten op het intakeformulier. Met geboortezorgprofessionals en Jeugdgezondheidszorg (JGZ) is gewerkt aan een signaleringsinstrument voor kwetsbaarheden in de zwangerschap: een digitale psychosociale vragenlijst die zwangere vrouwen voor het eerste consult bij de verloskundige invullen [10]. De bestaande verloskundige anamnese is uitgebreid met vragen die nodig zijn om te werken met de definitie van kwetsbaarheid en om brede sociale ondersteuning in te zetten. Ook andere professionals kunnen een rol spelen bij de signalering tijdens de zwangerschap, de kraamperiode en erna, zoals pedagogisch medewerkers (kinderopvang) en huisartsen. Voor deze professionals zijn nog geen concrete werkafspraken rond signalering vastgelegd in de Basisstructuur, maar dat is wel een ambitie voor de toekomst.

### Inzet zorgpaden

Alle zestien gemeenten hebben de landelijke digitale zorgpaden Kansrijke Start ingevuld. Deze zijn bedoeld om zorgverleners te helpen de juiste organisaties en contactpersonen binnen een gemeente te vinden op basis van een enkelvoudige hulpvraag van het gezin. Ze geven informatie over achttien verschillende thema’s (van huisvesting tot leefstijl) [11].

### JGZ-coördinator subregio

Vrouwen/gezinnen met niet-urgente kwetsbaarheden worden doorgeleid naar de JGZ-coördinator. In elk van de drie subregio’s is een coördinator aangesteld als persoonlijk aanspreekpunt voor professionals. Als de hulpvraag verder verkend moet worden, kan een prenataal huisbezoek door de JGZ worden gepland. Wanneer snel handelen gewenst is, kan een JGZ-coördinator meteen een doorverwijzing naar het Knooppunt Kansrijke Start inzetten.

### Prenataal huisbezoek JGZ

Na doorgeleiding brengt een jeugdverpleegkundige een prenataal huisbezoek. Doel is om de situatie en wensen van de zwangere in kaart te brengen en samen tot de best passende aanpak te komen. Mogelijkheden zijn het inzetten van Stevig Ouderschap/VoorZorg, zorg via een digitaal zorgpad of het gezin doorverwijzen naar het sociaal domein via het Knooppunt Kansrijke Start. Zo verbindt de JGZ het medisch en sociaal domein.

### Knooppunt Kansrijke Start

De Knooppunten Kansrijke Start vormen de schakel tussen de ondersteuningsvraag en het aanbod in het sociaal domein voor vrouwen/gezinnen in een kwetsbare situatie. Het Knooppunt is een multidisciplinair casuïstiekoverleg op gemeenteniveau met het gezin, de gemeente, JGZ, maatschappelijk werk en de aanmelder (indien deze dat wenst). De wensen en behoeften van het gezin zijn leidend. Doel is om snel en laagdrempelig de best passende steun beschikbaar te maken. Het sociaal netwerk van het gezin en/of andere professionals worden uitgenodigd op basis van betrokkenheid of expertise.

### Overleg zwangere in kwetsbare situatie

Vrouwen met urgente kwetsbaarheden (10% in Zuid-Limburg) worden doorgeleid naar het Overleg Zwangere in een Kwetsbare Situatie (OZKS), dat maandelijks plaatsvindt [8]. Vrouwen met verschillende niet-urgente kwetsbaarheden worden doorgeleid naar het OZKS of naar JGZ, afhankelijk van de aard van de problematiek (10% heeft drie of meer niet-urgente kwetsbaarheden) [8]. Het OZKS is een transmuraal overleg dat als doel heeft de medische en psychosociale zorg op elkaar af te stemmen en voor moeder en kind een veilige situatie te creëren. Naast het medisch domein (verloskunde, gynaecologie, kindergeneeskunde, psychiatrie) sluiten ook professionals uit het sociaal en publiek gezondheidsdomein aan (gemeente, JGZ, kraamzorg en sociale partners betrokken bij de casus). De behoeften en wensen van de zwangere of het gezin zijn ook in dit overleg leidend, maar de zwangere of het gezin sluiten hierbij niet aan (zoals wel gebeurt in het Knooppunt Kansrijke Start). Wanneer zorgen bestaan over de veiligheid van het ongeboren kind kan (anoniem) advies worden gevraagd aan Veilig Thuis en de Raad van de Kinderbescherming, die ook aanwezig zijn bij het OZKS. Dit geldt niet voor het gemeentelijke Knooppunt, waarbij deze partijen niet aansluiten.

## Projectorganisatie en implementatie Basisstructuur

Begin 2023 hebben de zestien Zuid-Limburgse gemeenten, de beide Verloskundig Samenwerkingsverbanden en de JGZ de Basisstructuur vastgesteld en zich daarmee gecommitteerd aan de samenwerking voor het leveren van passende zorg en ondersteuning aan zwangeren/(aanstaande) ouders in een kwetsbare situatie. Dit (bestuurlijke) draagvlak was een belangrijke mijlpaal en voorwaarde voor de verdere implementatie van de Basisstructuur in de gehele regio. Gemeenten, geboortezorg en JGZ zijn daarmee verantwoordelijk geworden voor de implementatie. Samen met GGD Zuid-Limburg en de Academie Verloskunde Maastricht hebben ze een werkgroep gevormd (zie bijlage 1 in de digitaal aanvullende content) die de volgende opdrachten kreeg:

1. de implementatie vormgeven, begeleiden, ondersteunen, borgen en evalueren;
2. zorg dragen voor het actualiseren en accuraat houden van de inhoud van de *Handleiding Kansrijke Start Zuid-Limburg*;
3. communicatie met de deelnemende partijen over de inhoud en implementatie.

Voor het implementeren van de Basisstructuur heeft de werkgroep een regionaal implementatieplan opgesteld. Drie projectleiders Kansrijke Start faciliteren en ondersteunen de implementatie, ook op gemeentelijk niveau. Een eerste stap in de implementatie in 2023 was het informeren van de betrokken partijen over de werkwijze van de Basisstructuur via presentaties tijdens de Lokale Coalities Kansrijke Start, discipline-overleggen van artsen en verpleegkundigen JGZ, en team-overleggen van onder andere het maatschappelijk werk, jeugdhulpaanbieders en ggz-aanbieders. Daarnaast werden alle verloskundigenpraktijken in Zuid-Limburg persoonlijk bezocht en vonden er gesprekken plaats met de ziekenhuizen in de regio. Dit bood inzicht in de kansen en belemmeringen voor het integreren van de Basisstructuur in de werkwijzen van professionals en organisaties. De gesprekken zijn in 2024 voortgezet, onder andere ook met huisartsenorganisaties, en er wordt gewerkt aan implementatieplannen per organisatie/beroepsgroep.

## Evaluatie

De werkgroep implementatie Basisstructuur evalueert het proces van implementatie, brengt de belemmerende en bevorderende factoren in beeld en doet voorstellen ter verbetering van de implementatie en opschaling naar heel Zuid-Limburg (zoals voor de digitale psychosociale vragenlijst en het Knooppunt Kansrijke Start). De resultaten uit de onderzoeksprojecten helpen daarbij.

Eerstelijnsverloskundigen in Zuid-Limburg geven de voorkeur aan een breed gespreksinstrument om vrouwen in een kwetsbare situatie te signaleren in plaats van een gevalideerd signaleringsinstrument (zoals Mind2care, R4U, ALPHA-nl). Onderzoek onder tien verloskundigenpraktijken laat zien dat verloskundigen met de ontwikkelde digitale psychosociale vragenlijst zwangeren in een kwetsbare positie goed in beeld krijgen en dat ze meer tijd overhouden voor het bespreken van knelpunten en wensen. De bereidheid onder zwangeren om de vragenlijst in te vullen is groot [10]. Uit het onderzoek komen ook succesfactoren naar voren die helpen bij de samenwerking tussen verloskundigen en JGZ, zoals persoonlijk contact en duidelijke werkafspraken, een eenduidig kader en een definitie voor kwetsbaarheid (wat samenwerking en gezamenlijke taal bevordert), duidelijk beschreven rollen, taken en verantwoordelijkheden, interprofessionele training, en voldoende tijd en financiën [8, 10]. Werken met het Knooppunt Kansrijke Start laat in drie pilotgemeenten (Kerkrade, Landgraaf en Vaals) zien dat het Knooppunt helpt om hulp beter af te stemmen op de behoeften van het gezin en dat gezinnen dit ook als waardevol ervaren.

Tegelijkertijd blijkt uit registraties dat het bereik van het Knooppunt en het prenataal huisbezoek JGZ nog laag is: het aantal aangemelde casussen blijft achter bij de verwachtingen op basis van het percentage (aanstaande) gezinnen in een kwetsbare situatie. Een van de genoemde verklaringen is dat (leden van) de beroepsgroepen onderling verschillen in de mate van betrokkenheid en de hoeveelheid kennis over (onderdelen uit) de Basisstructuur. Het implementeren en borgen van nieuwe werkwijzen op de werkvloer vergen tijd en geld, en worden belemmerd doordat organisaties en beroepsgroepen gebonden zijn aan domeinspecifieke wetgeving, financiering, structuren en beleid.

Uit de eerste resultaten van de onderzoeksprojecten komen verbeterpunten naar voren. Over het algemeen blijkt dat professionals positief zijn, maar dat er ook de wens is om de Basisstructuur verder uit te breiden naar de totale doelgroep van Kansrijke Start. Dus niet alleen focus op geboortezorg, maar ook aandacht voor ouders van kinderen van nul tot twee jaar, met signalering door de kinderopvang, huisartsen, het maatschappelijk werk, jeugdhulp of ggz-aanbieders.

Een ander verbeterpunt betreft de betrokkenheid van de doelgroep zelf bij de ontwikkeling van de Basisstructuur. Er zijn wel gesprekken geweest met een aantal gezinnen over hun ervaringen met de zorg en ondersteuning vanuit de Basisstructuur, maar het blijft lastig om deze gezinnen via de professionals bereid te krijgen om mee te werken aan een interview. Dit kan komen door terughoudendheid in verband met een mogelijk te grote belasting van het gezin, maar ook omdat de betrokken professionals de vertrouwensband tussen hen en de cliënt/patiënt niet willen schaden.

## Beschouwing

Op basis van de ontwikkeling en evaluatie van de Basisstructuur Kansrijke Start in Zuid-Limburg zijn enkele belangrijke geleerde lessen te noemen.

Ten eerste: investeer voldoende tijd in het elkaar leren kennen, heb oog voor elkaars belangen en verken de mogelijkheden voor samenwerking, aangejaagd door bijvoorbeeld projectleiders Kansrijke Start en leden van de Regionale Coalitie. Betrek vanaf het begin alle partijen uit het medisch, sociaal en publiek gezondheidsdomein bij de verschillende ontwikkelings-, besluitvormings- en implementatiefasen.

Ten tweede: zorg voor commitment van alle betrokken partijen uit het medisch, sociaal en publiek gezondheidsdomein voor het werken conform de Basisstructuur. Het realiseren van integrale domeinoverstijgende samenwerking binnen Kansrijke Start is mogelijk, maar is complex en tijdsintensief. Het GALA kent structurele financiële middelen voor Kansrijke Start, maar deze zijn niet toereikend. Daardoor moeten ook gemeenten en zorgverzekeraars financieel bijdragen, bijvoorbeeld bij het faciliteren van extra tijd voor professionals.

Ten derde: ontwikkel de ketenaanpak in cocreatie met de professionals die de werkwijzen moeten uitvoeren en sluit daarbij zo veel mogelijk aan bij de bestaande dagelijkse praktijk. Betrek hier ook de doelgroep zelf bij. In Zuid-Limburg zijn via professionals gezinnen benaderd en bereid gevonden om in gesprek te gaan over hun ervaringen met de zorg/ondersteuning. Tot op heden was deze aanpak echter onvoldoende om de doelgroep meer structureel te betrekken bij het ontwikkelen en implementeren van de Basisstructuur.

Ten vierde: besteed aandacht aan duurzame implementatie en monitor de impact van het werken conform de Basisstructuur. Maak een multidisciplinaire werkgroep verantwoordelijk voor de inhoud van de Basisstructuur, het opstellen van een implementatieplan en de monitoring ervan. Praktijkgericht onderzoek helpt om de doorontwikkeling en implementatie te ondersteunen.

Toekomstplannen voor duurzame implementatie van de Basisstructuur in Zuid-Limburg moeten er onder andere voor zorgen dat alle professionals de werkwijzen en afspraken uit de Basisstructuur daadwerkelijk kennen en gebruiken. Daarvoor zullen informerende en educatieve strategieën worden ingezet, zoals regionale (netwerk)bijeenkomsten, werkbezoeken en een website met alle relevante informatie en contactgegevens voor professionals. Ook wordt ingezet op interprofessionele deskundigheidsbevordering (e-learning, intervisie) voor adequate signalering en het wegnemen van barrières (handelingsverlegenheid) bij het afwegen van kwetsbaarheden en beschermende factoren, en het inzetten van sociale ondersteuning. Ten slotte wordt toegewerkt naar gepaste financiering, in samenwerking met gemeenten en zorgverzekeraars, voor de extra tijd die werken conform de Basisstructuur kost.

## Conclusie

In Zuid-Limburg leeft een gezamenlijke ambitie om alle kinderen een kansrijke start te geven, die voortkomt uit een breed besef van de noodzaak om gezondheidsachterstanden gezamenlijk aan te pakken. Met de inzet en het enthousiasme van de betrokken professionals en de commitment vanuit alle partijen zijn op regionaal niveau samenwerkingsafspraken gemaakt over het medisch, sociaal en publiek gezondheidsdomein heen: de Basisstructuur Kansrijke Start. Het programma Trendbreuk en de verschillende onderzoeksprojecten speelden hierbij een belangrijke en aanjagende rol. Duurzame implementatie van domeinoverstijgende ketensamenwerking vraagt om continue monitoring van kansen, belemmeringen en de impact van de samenwerking en implementatie. Op basis daarvan moeten passende implementatiestrategieën worden gekozen, die bijdragen aan structurele borging van de Basisstructuur in de werkwijzen van professionals en organisaties.

## Digitaal aanvullende content

Bijlage 1. Overzicht van partners uit de Regionale Coalitie Kansrijke Start Zuid-Limburg en hun rol in de opzet, uitvoering en implementatie van de Basisstructuur Kansrijke Start Zuid-Limburg
