# Supplementary material for: Integrated cross-sectoral collaboration for pregnant women and (expectant) parents in a vulnerable situation: the Solid Start integrated approach in South Limburg, the Netherlands
Source: TSG. 2025 Mar 25;103(2):37–43. [Article in Dutch] doi: 10.1007/s12508-025-00458-0 (PMC12185589; doi:10.1007/s12508-025-00458-0)
Supplement: Supplementary file 1 — Bijlage 1. Overzicht van partners uit de Regionale Coalitie Kansrijke Start Zuid-Limburg en hun rol in de opzet, uitvoering en implementatie van de Basisstructuur Kansrijke Start Zuid-Limburg [file 12508_2025_458_MOESM1_ESM.docx]

**Bijlage 1: *Overzicht van partners uit de Regionale Coalitie Kansrijke Start Zuid-Limburg en hun rol in de opzet, uitvoering en implementatie van de Basisstructuur Kansrijke Start Zuid-Limburg.***

| Organisatie in Regionale Coalitie Kansrijke Start | Rol in Basisstructuur |
| --- | --- |
| Academie Verloskunde Maastricht / Zuyd Hogeschool ^a^ | Opzet, implementatie en evaluatie |
| Burgerkracht Limburg | Adviseur |
| Geboortezorg Limburg (namens kraamzorg) ^a^ | Implementatie en uitvoering |
| Geboortezorg Consortium Limburg ^a^ | Implementatie |
| Gemeenten Zuid-Limburg (namens de 16 Zuid-Limburgse gemeenten):   - Gemeente Heerlen,   - Eén projectleider Kansrijke Start ^a^ - Gemeente Kerkrade ^a^ - Gemeente Sittard-Geleen - Gemeente Eijsden-Margraten ^a^ | Opzet, implementatie, uitvoering en financier |
| GGD Zuid-Limburg, met daar ondergebracht:   - Programmabureau Trendbreuk (voorzitter) ^a^ - Twee projectleiders Kansrijke Start ^a^ - Jeugdgezondheidszorg (JGZ) Zuid-Limburg ^a^ - Coördinatoren van programma’s en interventies (Rookvrije Start, Nu Niet Zwanger, VoorZorg, Stevig Ouderschap) - Academische Werkplaats Publieke Gezondheid Mosa | Opzet en implementatie  Opzet en implementatie  Opzet, implementatie en uitvoering  Uitvoering  Evaluatie |
| Humankind (namens kinderopvang) | Uitvoering |
| Maastricht Universitair Medisch Centrum+ (MUMC+) ^a^ | Opzet, implementatie en uitvoering |
| Pharos | Adviseur |
| Provincie Limburg *(agendalid)* ^b^ | Financier |
| Verloskundig Samenwerkingsverbanden (VSV)   - VSV Samen Zuyd ^a^ - VSV Maastricht-Heuvelland ^a^ | Implementatie en uitvoering  Implementatie en uitvoering |
| Zorgverzekeraar CZ *(agendalid)* ^b^ | Financier |
| Zuyderland Medisch Centrum ^a^ | Implementatie en uitvoering |

^a^ Partners die deel uitmaken van de werkgroep implementatie Basisstructuur Kansrijke Start Zuid-Limburg

^b^ Zorgverzekeraar CZ en de Provincie Limburg zijn beide tevens bestuurlijk vertegenwoordigd in de Stuurgroep Trendbreuk (hierin zitten naast CZ en Provincie Limburg ook vertegenwoordigers van de gemeenten, MUMC+, GGD Zuid-Limburg, Welzijnswerk, Onderwijs en kinderopvang). In de Stuurgroep Trendbreuk vindt bestuurlijke aansturing van de Trendbreuk-ambitie plaats over alle levensfasen binnen Trendbreuk, waaronder ook Kansrijke Start.
